# Draft Genome Sequence of *Serratia fonticola* LMG 7882^T^ Isolated from Freshwater

**DOI:** 10.1128/genomeA.00971-13

**Published:** 2013-11-21

**Authors:** Adriana Ribeiro Carneiro, Rommel Thiago Jucá Ramos, Rafael Azevedo Baraúna, Pablo Henrique de Sá, Diogo Marinho Almeida, Silvanira Barbosa, Anabela Pereira, Artur Alves, Conceição Egas, António Correia, Isabel Henriques, Artur Silva

**Affiliations:** Instituto de Ciências Biológicas, Universidade Federal do Pará, Belém, Pará, Brazila; Department of Biology and CESAM, University of Aveiro, Campus Universitário de Santiago, Aveiro, Portugalb; Biocant-Biotechnology Innovation Center, Cantanhede, Portugalc

## Abstract

*Serratia fonticola* is a Gram-negative bacterium with a wide distribution in aquatic environments. On some occasions, it has also been regarded as a significant human pathogen. In this work, we report the first draft genome sequence of an *S. fonticola* strain (LMG 7882^T^), which was isolated from freshwater.

## GENOME ANNOUNCEMENT

The genus *Serratia* comprises Gram-negative, rod-shaped, facultative anaerobes in the family *Enterobacteriaceae*. Members of this genus have been associated with a variety of habitats, such as water, soil, insects, plants, and humans (1). The genus includes strains that cause important infections in humans and other animals, particularly strains belonging to the species *Serratia marcescens* and the so-called *Serratia liquefaciens*-like species (1). *Serratia fonticola* was described in 1979 by Gavini et al. and is a ubiquitous inhabitant of aquatic environments, particularly freshwater. This species differs considerably from other *Serratia* species in terms of its DNA G+C content and also because most strains ferment d-dulcitol and do not produce DNase and gelatinase. For these reasons, its classification within the *Serratia* genus has been questioned (2). However, using 16S rRNA gene sequence analysis, *S. fonticola* has been found to belong to this genus (1). The first human infection caused by *S. fonticola* was reported in 1989 in a patient who had been involved in a car accident (3). On that occasion, the bacterium was recovered in pure culture from a purulent leg abscess and from a blood culture bottle. Since then, *S. fonticola* has been regarded as a significant human pathogen to which a variety of infections have been attributed (4–7). To our knowledge, there is no record of an *S. fonticola* genome sequence in the public databases.

*S. fonticola* LMG 7882 (ATCC 29814) is the type strain of the species and was isolated from freshwater (8). The draft genome sequence of this strain is presented here.

Genome sequencing was carried out using the Genome Sequencer FLX 454 (Roche) with a fragment library, and 72,753,131 bp was produced. The sequences were extracted from the .sff file with the script “sff_extract_0_2_13” (http://bioinf.comav.upv.es/sff_extract/). The reads were evaluated using Quality Assessment Long Reads (9) to remove low-quality reads using an average Phred score of 20. After this process, a total of 249,830 reads (69,827,222 bp) remained, representing a coverage of ~12×.

A *de novo* assembly approach was conducted using the software MIRA (10) and produced 427 contigs. This set of sequences was reassembled using SeqMan NGen version 11 to identify and remove redundant sequences, curate conflicts between the alignments, and extend the sequences that present similarities in their ends. The final draft assembly contained 363 contigs. The estimated size of the genome is 5,895,861 bp, with an N_50_ of 49 kb and a G+C content of 54%. Annotation using the Rapid Annotations using Subsystems Technology (RAST) server (11) predicted 5,214 coding sequences (CDSs), 74 tRNAs, and 5 rRNAs.

The draft genome sequence of *S. fonticola* LMG 7882^T^ will enable further studies with the aim of understanding the ecology, virulence potential, and antibiotic resistance of this species.

### Nucleotide sequence accession numbers.

The *S. fonticola* LMG 7882^T^ draft genome sequence has been deposited in GenBank under the accession no. AVAH00000000. The version described in this paper is version AVAH01000000.

## References

[1] Mahlen SD 2011 *Serratia* infections: from military experiments to current practice. Clin. Microbiol. Rev. 24:755–791 21976608 10.1128/CMR.00017-11 PMC3194826

[2] Farmer JJ III Boatwright KD Janda JM 2007 *Enterobacteriaceae*: introduction and identification, p 649–669 *In* Murray PR Baron EJ Jorgensen JH Landry ML Pfaller MA (ed), Manual of clinical microbiology, 9th ed. ASM Press, Washington, DC

[3] Bollet C Gainnier M Sainty JM Orhesser P De Micco P 1991 *Serratia fonticola* isolated from a leg abscess. J. Clin. Microbiol. 29:834–835 1890188 10.1128/jcm.29.4.834-835.1991 PMC269884

[4] Gorret J Chevalier J Gaschet A Fraisse B Violas P Chapuis M Jolivet-Gougeon A 2009 Childhood delayed septic arthritis of the knee caused by *Serratia fonticola*. Knee 16:512–514 19401267 10.1016/j.knee.2009.02.008

[5] Kunimoto D Rennie R Citron DM Goldstein EJ 2004 Bacteriology of a bear bite wound to a human: case report. J. Clin. Microbiol. 42:3374–3376 15243122 10.1128/JCM.42.7.3374-3376.2004 PMC446265

[6] Pfyffer GE 1992 *Serratia fonticola* as an infectious agent. Eur. J. Clin. Microbiol. Infect. Dis. 11:199–200 1396739 10.1007/BF01967080

[7] Soler T Samson E Hernandez T Hervé V Revel T 2000 *Serratia fonticola*, opportunistic pathogen: report of a case in immunocompromised patient. Med. Mal. Infect. 30:599–600

[8] Gavini C Ferragut D Izard P Trinel A Leclerc H Lefebvre B Mossel DAA 1979 *Serratia fonticola*, a new species from water. Int. J. Syst. Bacteriol. 29:92–101

[9] Ramos RT Carneiro AR Soares SD Santos AR Almeida S Guimarães L Figueira F Barbosa E Tauch A Azevedo V Silva A 2013 Tips and tricks for the assembly of a *Corynebacterium pseudotuberculosis* genome using a semiconductor sequencer. Microbiol. Biotechnol. 6:150–156 10.1111/1751-7915.12006 PMC3917457 23199210

[10] Chevreux B Wetter T Suhai S 1999 Genome sequence assembly using trace signals and additional sequence information, p 45–56 *In* Computer science and biology: proceedings of the German Conference on Bioinformatics. Research Center for Biotechnology (GCB), Braunschweig, Germany

[11] Aziz RK Bartels D Best AA DeJongh M Disz T Edwards RA Formsma K Gerdes S Glass EM Kubal M Meyer F Olsen GJ Olson R Osterman AL Overbeek RA McNeil LK Paarmann D Paczian T Parrello B Pusch GD Reich C Stevens R Vassieva O Vonstein V Wilke A Zagnitko O 2008 The RAST server: rapid annotations using subsystems technology. BMC Genomics 9:75. 10.1186/1471-2164-9-75 18261238 PMC2265698

